# Polyaniline/Reduced Graphene Oxide Composites for Hole Transporting Layer of High-Performance Inverted Perovskite Solar Cells

**DOI:** 10.3390/polym13081281

**Published:** 2021-04-14

**Authors:** Jae Woong Jung, Seung Hwan Son, Jun Choi

**Affiliations:** 1 Department of Advanced Materials Engineering for Information and Electronics, Integrated Education Institute for Frontier Materials (BK21 Four), Kyung Hee University, 1732 Deogyeong-daero, Giheung-gu, Yongin-si 446-701, Gyeonggi-do, Korea; wodndwjd@khu.ac.kr; 2 Advanced Textile R&D Department, Korea Institute of Industrial Technology (KITECH), Ansan-si 426-910, Gyeonggi-do, Korea; maru072@kitech.re.kr; 3 Human Convergence Technology R&D Department, Korea Institute of Industrial Technology (KITECH), Ansan-si 426-910, Gyeonggi-do, Korea

**Keywords:** polyaniline, perovskite solar cells, hole transport layer

## Abstract

We herein address the optoelectronic properties of polyaniline composite films with graphene oxide and reduced graphene oxide as a hole transport layer in inverted perovskite solar cells. The composite films exhibited enhanced electrical conductivity and suitable energy level matching with CH_3_NH_3_PbI_3_ for efficient hole extraction/transport than the pristine polyaniline film, which thus can deliver improved photovoltaic properties of device. The composite film-based devices exhibited maximum efficiency of 16.61%, which is enhanced by 21.6% from the device with the pristine polyaniline hole transport layer (efficiency = 13.66%). The reduced graphene oxide-based composite film also achieved improved long-term operative stability as compared to the pristine polyaniline-based device, demonstrating a great potential of reduced graphene oxide/polyaniline composite hole transport layer for high performance perovskite solar cells.

## 1. Introduction

Organometallic trihalide perovskites have been regarded as a representative next-generation photoactive material for photovoltaic applications, due to their unique optoelectronic properties such as intense light absorptivity, excellency in carrier transport and diffusion length, diversity in structure, and proper optical band gap [1,2]. The photovoltaic property of perovskite solar cells (PSCs) has been advanced by materials engineering, interface modification, and device engineering, which have achieved power conversion efficiency (PCE) of >25% over the last five years [3,4,5,6]. In general, the most well-established device structure of PSCs with high efficiency is a regular structure in which the transparent conductive oxide (TCO) cathode is modified with the metal oxide-based electron transport layer (ETL) (e.g., TiO_2_ or SnO_2_) for efficient electron transport from the perovskite absorber layer. However, it would not be a suitable structure for flexible devices because a high-temperature sintering process is required for good electrical properties of the metal oxide-based ETLs [7]. The inverted device is attracting much attention as an alternative device architecture of the conventional regular devices due to the low-temperature processability of a wide range of interfacial layer materials [8]. In the inverted structured device, the hole transport layer (HTL) is deposited onto the TCO anode to facilitate the hole transport from the perovskite absorber, while the ETL should be present between the interface of the perovskite layer and the metal anode [9].

Poly(3,4-ethylenedioxythiophene):polystyrene sulfonate (PEDOT:PSS) is one of the most successful solution-processed HTL materials in organic optoelectronic devices, because it can provide a smooth and uniform surface, appropriate work function of the ITO electrode, and favorable surface tension for deposition of the perovskite absorber precursors [10]. However, there still remain issues such as high acidity of the PEDOT:PSS solution that would corrode the ITO electrode and perovskite absorber, which deteriorates the operative stability of the devices. More importantly, it has been reported that the work function of PEDOT:PSS (~4.4 eV) is not well balanced with the energy levels of perovskite absorbers (~−3.9 and −5.4 eV for conduction and valence band, respectively, for CH_3_NH_3_PbI_3_), which may limit the full potential of open-circuit voltage (*V*_OC_) and resultant PCEs of PSCs [11]. To resolve these issues, HTL engineering has been vigorously studied for the last decade. Among the inorganic semiconducting materials, the *p*-type oxides (e.g., nickel oxide) have been widely used in the inverted device of PSCs. Recent reports on the *p*-type oxide-based HTLs delivered high PCEs up to 20% in the planar inverted PSCs [12,13]. However, the *p*-type oxides generally require a high temperature sintering process to achieve high electrical properties, so it could not be applied in plastic substrate [14]. As an alternative, conjugated *p*-type polymers, such as poly(triaryl amine) (PTAA), have been studied as a promising HTL in the inverted PSCs. However, the perovskite precursors in polar solvents could not be easily processed onto the hydrophobic surface of PTAA [15]. Thus, a novel polymeric HTL that exhibits better energy level alignment and processing compatibility is essential as a replacement of PEDOT:PSS of the inverted PSC.

Polyaniline (PANI) is another representative *p*-type conductive polymer that has been widely used for organic electronics [16]. Recently, inverted devices employing the PANI-based HTLs have been studied due to their high electrical conductivity, well-established synthetic routes, and environmental/optical/chemical stabilities [17]. Li et al. reported on the PANI thin film prepared by electropolymerization, and its application in the PSCs [18]. Shin et al. synthesized PANI nanoparticles to use in HTL of PSCs [19]. The solution-processed PANI doped with PSS has also been reported as a replacement of PEDOT:PSS in inverted PSCs [20]. Very recently, a composite film of PEDOT:PSS/ PANI/graphene oxide (GO) has accomplished balanced energy level matching and increased electrical conductivity, which demonstrated an excellent HTLs in the inverted PSCs with a PCE of 18.12% [21]. Huang et al. also demonstrated an RGO/PEDOT:PSS composite HTL in inverted PSCs with a PCE of 10.5% [22]. Other types of RGO-based composites, such as RGO/MoO_3_, have also been utilized as a HTL in the planar inverted PSCs [23]. Thus, a nanocomposite of graphene nanomaterials (GO or RGO) can be a promising candidate of HTL in the inverted planar PSCs for achieving high photovoltaic properties. In particular, PANI could be a good polymer matrix for optimizing the optoelectronic properties of the composite HTLs. However, there is rare reports on the nanocomposites of PANI with reduced graphene oxide (RGO), while RGO can be regarded as an interesting nanomaterial in optoelectronics due to its higher electrical conductivity, less surface defects, and larger work function than GO [24,25].

In this study, we prepared two PANI/graphene nanocomposite films (PANI/GO and PANI/RGO), which were prepared from the water-soluble, self-doped PANI. In addition, we comprehensively studied the optoelectronic properties of PANI/graphene nanocomposite films to examine their potential as a novel HTL in the planar heterojunction PSCs in inverted structure. The optoelectronic properties and physical characteristics of PANI/GO and PANI/RGO were compared, which concluded better energy level matching and comparable electrical conductivity compared to the pristine PANI. The optimized PANI-based nanocomposite HTLs achieved PCEs of 14.11% and 16.61% with GO and RGO, respectively, which were higher than the pristine PANI-based HTL (PCE = 13.66%) in the *p-i-n* planar heterojunction device with a CH_3_NH_3_PbI_3_ perovskite absorber. More importantly, the composite HTLs exhibited negligible *J-V* hysteresis and high long-term stability in ambient condition without encapsulation, which promises the potential of PANI/graphene composite for applications in perovskite optoelectronics.

## 2. Materials and Methods

### 2.1. Materials

Poly(styrenesulfonic acid)-graft-polyaniline (PSSA-*g*-PANI) was synthesized as reported elsewhere [26]. The molar ratio of aniline to styrenesulfonic acid of PSSA-*g*-PANI was set to 0.2 to optimize the electrical property and water solubility. The GO was prepared by a modified Hummers method [27]. RGO was obtained using *p*-toluenesulfonyl hydrazine as a reducing agent as reported elsewhere [28]. PbI_2_ (99.998%, Sigma-Aldrich, St. Louis, MO, USA), Methylammonium iodide (Greatcellsolar, Queanbeyan, Australia), PC_61_BM (99%, Nano-C, Westwood, MA, USA), bathocuproine (BCP, 99%, Sigma-Aldrich), were obtained from commercial source, and used as received without further purification. Indium tin oxide (ITO)-coated glass substrates (sheet resistance = 15 ohm/sq) were provided by Omniscience.

### 2.2. Preparation of PANI/Graphene Nanocomposite Solution

After 10 mg of graphene nanomaterials (GO or RGO) were dispersed in 5 mL of deionized water in bath-type sonicator for 1 h, GO (0.5 mL) or RGO (0.25 mL) dispersions were added into 1 mL of PSSA-*g*-PANI aqueous solution (2 wt%), which set the concentration of GO/PSSA-*g*-PANI and RGO/ PSSA-*g*-PANI to be 5% and 2.5%, respectively. The composite solutions were stirred overnight and then sonicated for 30 min before use.

### 2.3. Device Fabrication and Characterization

The ITO-coated glass substrates (sheet resistance = 20 Ω sq^−1^) were cleaned sequentially with water, acetone, and isopropanol under sonication for 20 min, and treated with UV-O_3_. The HTLs (PSSA-*g*-PANI or nanocomposites) were deposited by spin-coating onto ITO-coated glass (3000 rpm, 60 s), and then the films were annealed at 150 °C for 30 min in ambient atmosphere. After cooling the substrate to room temperature, the perovskite precursor solution (461 mg of PbI_2_, 159 mg of methylammonium iodide, and 78 mg of dimethyl sulfoxide (molar ratio 1:1:1) in 600 mg of dimethylformamide) was spin-coated via two steps (500 rpm for 5 s and then 5000 rpm for 25 s). After 7 s, 400 µL of anhydrous chlorobenzene (0.2 mL) was poured onto the substrate during spin coating. The films were annealed at 100 °C for 10 min. PC_61_BM and BCP were deposited by spin-coating the solution (1 wt% in chlorobenzene and 0.2 wt% in methanol for PC_61_BM and BCP, respectively). Finally, 100 nm of Ag was evaporated under high vacuum (<5 × 10^−6^ Torr). The device area was defined as 10 mm^2^ by the shadow mask.

### 2.4. Characterizations

The transmittance of the HTL film was studied by a UV-Vis-NIR photospectrometer (Carry5000, Agilent, Santa Clara, CA, USA). The photoelectron spectroscopy measurements were carried out using AXIS ultra-delay line detector (DLD) (Kratos, UK) equipped with He I gas (21.22 eV) as a UV source. The fluorescence of the perovskite absorber was measured using a spectrofluorometer (FS5, Edinburgh instruments). The nanostructured surface of the film was observed by an atomic force microscope (AFM) (CoreAFM, Nanosurf, Liestal, Switzerland) and field-emission scanning electron microscopes (FE-SEMs) at the Core Facility Center for Analysis of Optoelectronic Materials and Devices of Korea Basic Science Institute (KBSI) (Supplementary Material). The X-ray diffraction of the perovskite absorber was characterized by X-ray diffraction system (Miniflexi300, Rikagu, Tokyo, Japan). The photovoltaic property of the device was obtained by measuring the current density–voltage (*J-V*) curves using a parameter analyzer (4200-SCS, Keithley, Cleveland, OH, USA) under 100 mW cm^−2^ illumination (AM 1.5G), which was calibrated using an NREL-certified photodiode. The voltage scan rate was 20 mV s^−1^ in forward/reverse scan direction, and the scan range was 0 to 1.2 V. The photoactive layer of the device was defined by a shadow mask upon evaporation of Ag electrode (active area = 10 mm^2^). The external quantum efficiency (EQE) of the devices was measured using a lock-in amplifier system, which records the short-circuit current density (*J*_SC_) under chopped monochromatic light.

## 3. Results and Discussion

**Scheme 1:**
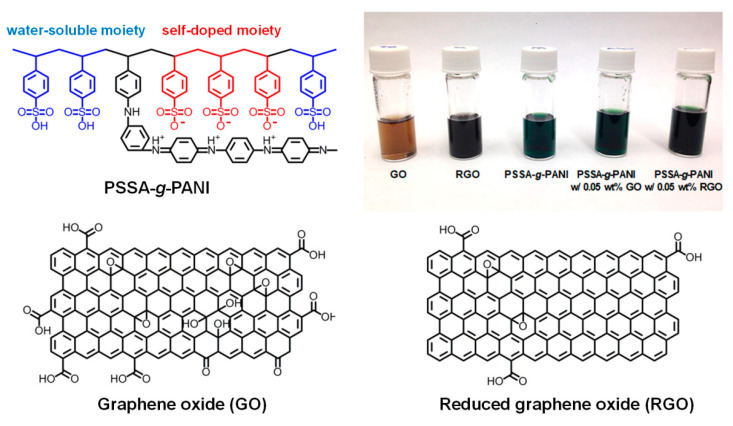
Chemical structure and aqueous solution of the materials used in this work.

Scheme 1 displays the molecular structure of the composite materials used in this work. PSSA-*g*-PANI is a water-soluble and self-doped PANI copolymer grafted with PSSA, where PSSA is a preferred polymer template for solubilizing and stabilizing the doped PANI. As a result, PSSA-*g*-PANI possesses an order of magnitude higher conductivity and lower work function as compared to PEDOT:PSS, thus can be a promising HTL for optoelectronic devices [29]. To explore the optoelectronic properteis of the PANI/graphene nanocomposites, two types of graphene nanomaterials (GO and RGO) were mixed with PSSA-*g*-PANI (Figure S1 for TEM images for graphenes in solution; Figure S2 for AFM image for graphenes in film). Two solutions of PSSA-*g*-PANI/GO (denoted as PANI/GO, hereafter) and PSSA-*g*-PANI/RGO (denoted as PANI/RGO, hereafter) were well soluble in DI water, as shown in Scheme 1. The solutions were stable for several weeks without precipitation.

**Figure 1:**
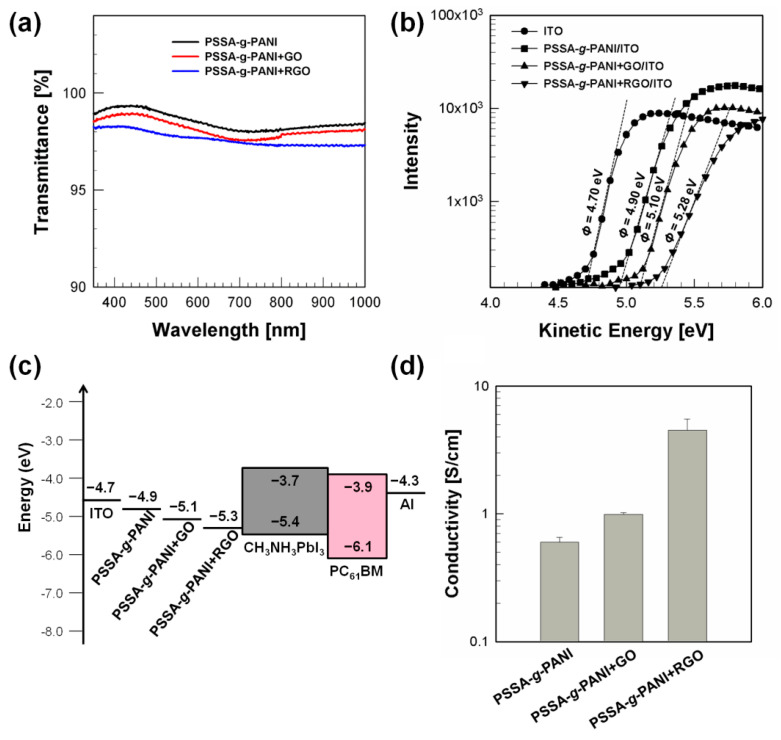
(**a**) Transmittance spectra, (**b**) UPS kinetic energies, (**c**) energy level diagram, and (**d**) electrical conductivities of the composite films in PSCs.

The PANI nanocomposite films were prepared by the spin coating, and the film thickness was set to 30–50 nm which has been optimized for efficient hole transport in organic solar cells [29]. The PANI thin film exhibited a very uniform surface with a small roughness less than 5 nm, but a textured surface was observed in the composite films, as shown in AFM images, which is thought to be due to the GO or RGO element in the composite films (Figure S1). The optical transmittance of PANI and the corresponding nanocomposites films were compared, as shown in Figure 1a. The PSSA-*g*-PANI films exhibited high transmittance over the UV-Vis-NIR range, but the composite films exhibited rather lower optical transmittance due to the broad absorption properties of the graphene nanomaterials. Despite the PANI/RGO composite film possessing the least transmittance as compared to pristine PANI or PANI/GO films, the overall transmittance of the three films was >96% in the range of 300 to 1000 nm, thus it is expected that there would be no significant decrease in photovoltaic performance originating from the parasitic absorption loss in the graphene nanocomposite films.

The work function of HTL plays a key role in determining the *V*_OC_ of PSCs, because the driving force of exciton dissociation and charge transport is dependent on the difference of the work functions of the anode and cathode. Figure 1b shows the Fermi level of the ITO, PANI, and the nanocomposite films measured by ultraviolet photoelectron spectroscopy (UPS). The work function of ITO-coated glass was 4.7 eV, and it was increased to 4.9 eV as the surface of ITO was modified with PSSA-*g*-PANI. In the case of the PANI/graphene nanocomposite films, the work function was gradually increased to 5.1 and 5.3 eV with GO and RGO, respectively. The valence band edge of CH_3_NH_3_PbI_3_ is ~−5.4 eV, so the enlarged work function of the PANI nanocomposite film is beneficial in energy level alignment at the interface of HTL and the perovskite absorber, which would lead to facilitated hole transport and enhanced photovoltage generation [30] (Figure 1c). The electrical conductivity is another pivotal factor for the photovoltaic properties of PSCs, because the photocurrent of PSCs is dependent on the electrical conductivity of the interfacial layers [31]. As displayed in Figure 1d, the PANI/GO composite film possesses a higher electrical conductivity than that of pristine PANI film, and the PANI/RGO composite film exhibited electrical conductivity almost an order of magnitude higher than the PANI film. The high electrical conductivity of the PANI/graphene composite would contribute to enhanced hole transport and reduced charge recombination at the interface of the device.

**Figure 2:**
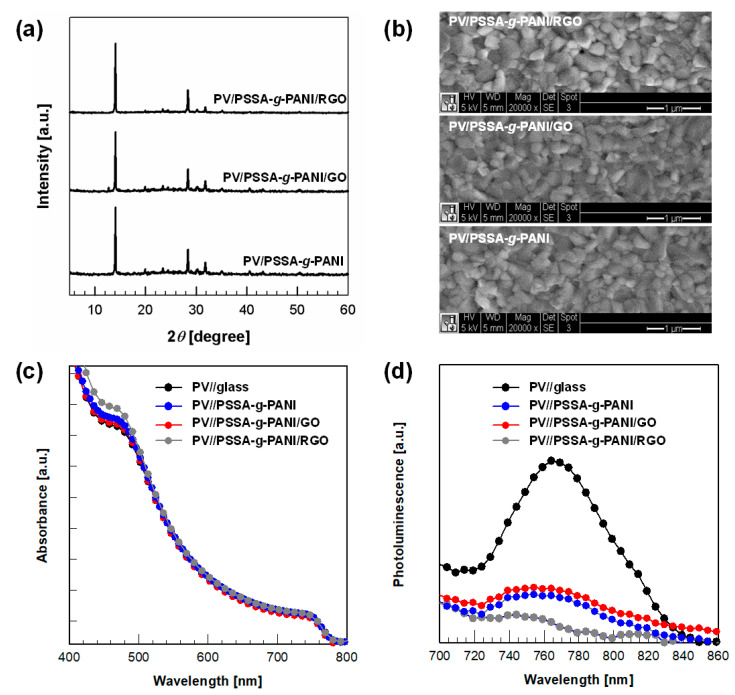
(**a**) XRD diffractograms, (**b**) FE-SEM images, (**c**) UV-Vis absorption spectra, and (**d**) steady state PL emission of the CH_3_NH_3_PbI_3_ layer on the composite HTLs. Scale bar of FE-SEM image denotes 1000 nm.

The morphological characteristics of the perovskite absorber, such as crystallinity, surface coverage, and roughness, are critical for optimization of the photovoltaic properties of PSCs. The film crystallinity of the perovskite absorber onto the composite HTLs was studied by X-ray diffractograms (XRD), as shown in Figure 2a. The XRD patterns of CH_3_NH_3_PbI_3_ films grown on three HTLs showed the tetragonal for CH_3_NH_3_PbI_3_ perovskite structure, with the Bragg peaks at 2θ = 14.1°, 28.2°, and 31.8°, which are assigned to (110), (220), and (310) planes, respectively, as reported elsewhere [32,33]. The intensities of the scatterings were comparable in three HTL films without any peak shift peaks. The surface morphology of the perovskite absorbers grown on different HTLs was further investigated by FE-SEM. As shown in Figure 2b, homogeneous and smooth surface morphology with a comparable grain size (200–300 nm) was found in the perovskite absorber films regardless of the composite composition. When the absorption properties of the perovskite films were further studied by UV-Vis absorption spectra, almost the same spectra with an absorption shoulder and an absorption onset at ~755 and ~780 nm, respectively, were observed, indicating that crystalline perovskite films were grown on the HTL films regardless of their composite composition (Figure 2c). The charge extraction capability of the PANI nanocomposite HTLs was further studied by steady-state PL. Figure 2d displays the PL emission spectra of the CH_3_NH_3_PbI_3_ films, which was centered at 766 nm. As the HTLs were employed, the PL of the perovskite absorber was significantly quenched, and the quenching efficiency was more enhanced in the PANI/graphene composite films than the pristine PSSA-*g*-PANI. This represents the well-crystallized perovskite thin films growth on the PANI/graphene HTL films. It is noted that the least PL intensity was observed from the perovskite absorber grown onto the mixed film of PSSA-*g*-PANI and RGO, which reveals the highest quenching efficiency of the PANI/RGO nanocomposite HTL.

**Figure 3:**
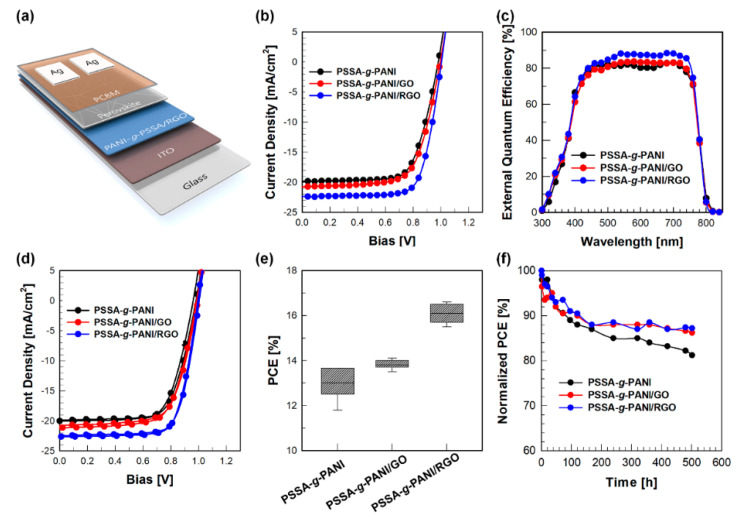
(**a**) Schematic device structure, (**b**) *J-V* curves under 1-sun illumination, (**c**) EQE spectra, (**d**) *J-V* curves under 1-sun illumination with different scan directions, (**e**) statistical analysis of PCEs, and (**f**) PCE decay under ambient storage of the PSCs with the composite HTLs.

The influence of PANI/graphene nanocomposite-based HTLs on the device performance of PSCs was examined by fabricating the inverted devices with a planar heterojunction (*p-i-n*) structure (Figure 3a). The device structure is ITO/HTLs/CH_3_NH_3_PbI_3_/PC_61_BM/BCP/Ag, where PC_61_BM and BCP are used as an electron transport layer and a cathode modifier, respectively. Figure 3b displays the *J-V* characteristics of the devices employing the PANI nanocomposite HTLs, and the corresponding parameters are listed in Table 1. The device with a PSSA-*g*-PANI film delivered a PCE up to 13.66%, with a *V*_OC_ of 0.99 V, a *J*_SC_ of 19.86 mA cm^−2^, and a fill factor (*FF*) of 0.69. In the case of PANI/GO-based HTL, the device showed a slightly improved PCE of 14.11% with a *V*_OC_ of 1.00 V, a *J*_SC_ of 20.78 mA cm^−2^, and an *FF* of 0.68. The PCE was further increased to 16.61% as RGO was mixed with PANI in the HTL, which was comparable to those of PEDOT:PSS-based inverted PSCs [34]. The improved PCE for the PANI/RGO-based HTL is mainly originated from the enhancement of the *J*_SC_ (22.38 mA cm^−2^) and *FF* (0.74), which were increased by 12.7% and 7.2%, respectively. The enhancement in *J*_SC_ and *FF* would be attributed to higher conductivity and favorable energy level matching of the composite HTLs with the perovskite absorber, as compared to the pristine PANI film. The contribution of the better energy level matching of the interface at the HTL and perovskite absorber was further confirmed by the significantly reduced series resistance (*R*_s_) of the devices (Table 1).

The EQE for the device with a PANI/RGO exhibited the highest photon-to-electron conversion capability in the range 400–750 nm, which reveals superior electrical properties of PANI/RGO among the PANI-based HTLs studied in this work (Figure 3c). The integrated EQE values are well matched with the measured *J*_SC_ under illumination. It is also noted that the hysteresis behavior was negligible in three devices, but the PANI/RGO HTL delivered the least hysteresis index (HI) (Table S1), which reveals the reliability and superiority of the photovoltaic performance of the PANI/RGO film rather than other HTLs (Figure 3d). The statistical distributions of PCEs for more than 15 devices in each HTL were summarized in Figure 3e. A substantial enhancement in the PCEs in the PANI/RGO-based HTL is obviously present. It is interesting that the PANI/graphene nanocomposite HTLs delivered less deviation of PCEs, as compared to the pristine PANI HTL, which reveals the reliability of the photovoltaic performance of PSCs which could be correlated with the higher conductivity of HTLs.

To investigate the long-term stability of the devices, we monitored the PCE decay of the devices with different HTLs for 500 h in ambient condition (relative humidity ~40%, temperature ~20 °C) without encapsulation. Figure 3f shows the degradation of the PCE as a function of storage time. After 500 h in ambient atmosphere, the device with PSSA-*g*-PANI HTL exhibited 82% value of the initial efficiency, while the composite HTLs retained more than 86% of the initial PCEs. Specifically, PANI/RGO HTL retained 87% of the initial PCE value after 500 h in ambient storage, indicating the graphene nanocomposites could provide better long-term stability of the planar inverted PSCs. Although the exact reasons for the better stability are still unclear, it is expected that the addition of graphene into the hydrophilic PSSA-*g*-PAN reduces the hydrophilic properties, which could contribute to resistance against the moisture invasion into the devices. It cannot be readily concluded that the stability of the devices studied in this work is better than that of other structures (e.g., normal meso structure), but it is more stable than the stability of the typical PEDOT:PSS-based inverted device without encapsulation [35]. Thus, we believe that the graphene composite HTL would lead to enhanced device stability under long-term operation, which should play an important role in the long-term stability of PSCs [36].

## 4. Conclusions

In summary, this study demonstrated the utilization of PANI/graphene nanocomposite films as a HTL of the inverted PSCs. The PANI/graphene nanocomposite HTLs exhibited favorable electrical properties, including high conductivity and better energy level matching to the perovskite absorber (CH_3_NH_3_PbI_3_), despite a little loss in optical transmittance by graphene. As a result, the improved optoelectronic features in the PANI nanocomposite films led to superb hole extraction capability, as compared to the pristine PANI HTL. The inverted PSCs fabricated with the PANI/graphene nanocomposites achieved a PCE up to 14.11% for PANI/GO HTL and 16.61% for PANI/RGO HTL with negligible *J-V* hysteresis. The long-term stability of the devices in ambient condition was also improved in the PANI/graphene nanocomposite HTL-based devices, retaining >86% of the initial efficiency without encapsulation after 500 h. The results studied in this work suggest that the PANI/graphene-based nanocomposite films can be a promising candidate of HTLs to achieve high efficiency and ambient stability of PSCs. We also expect that new types of composite films, by using not only graphene-based composite materials but also other metal/ceramic materials with suitable physical/chemical/electrical properties, will accelerate the efficiency and stability of PSCs.

## Figures and Tables

**Table 1:** Photovoltaic parameters of devices with varied HTL composites under 1-sun illumination.

Hole Transport Layer	*V*_OC_ [V]	*J*_SC_ [mA/cm^2^]	*FF*	PCE [%]	*R*_s_ [Ω]
PSSA-*g*-PANI	0.99	19.86	0.69	13.66	2.68
PSSA-*g*-PANI/GO	1.00	20.78	0.68	14.11	1.74
PSSA-*g*-PANI/RGO	1.01	22.38	0.74	16.61	0.66

## Data Availability

The data presented in this study are available on request from the corresponding author.

## Supplementary Materials

The following are available online at https://www.mdpi.com/article/10.3390/polym13081281/s1, Figure S1: TEM images of graphene oxide, Figure S2: AFM images of graphene oxide.
