# Supplementary material for: Polyaniline/Reduced Graphene Oxide Composites for Hole Transporting Layer of High-Performance Inverted Perovskite Solar Cells
Source: Polymers (Basel). 2021 Apr 14;13(8):1281. doi: 10.3390/polym13081281 (PMC8071048; doi:10.3390/polym13081281)
Supplement: Supplementary file 1 [file polymers-13-01281-s001.pdf]

polymers

MDPI

Supplementary Material: Polyaniline/Reduced Graphene Oxide Composites for Hole Transporting Layer of High-Performance Inverted Perovskite Solar Cells

Jae Woong Jung, Seung Hwan Son, and Jun Choi

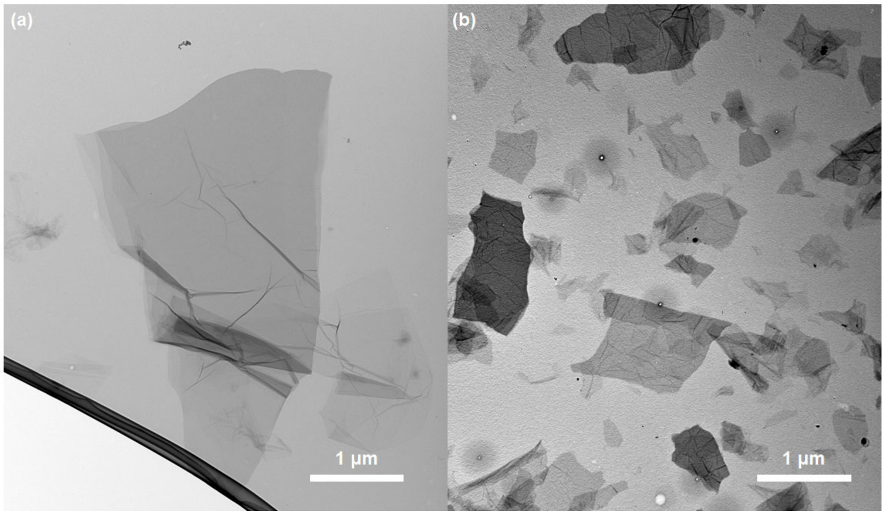

Figure S1. TEM images of graphene oxide (a) and reduced graphene oxide (b).

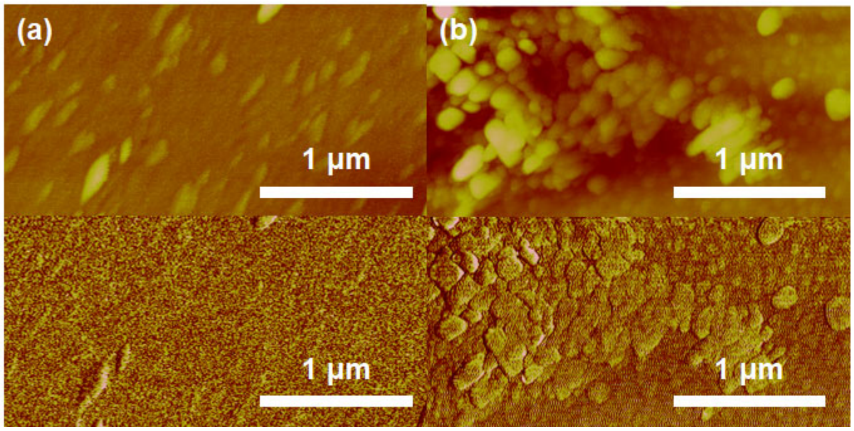

Figure S2. AFM images of graphene oxide (a) and reduced graphene oxide (b) deposited on glass substrate. (top: height; down: phase).
